# A Nationwide Mystery Caller Evaluation of Oral Emergency Contraception Practices from German Community Pharmacies: An Observational Study Protocol

**DOI:** 10.3390/healthcare9080945

**Published:** 2021-07-26

**Authors:** Christian Kunow, Moulika Aline Bello, Laura Diedrich, Laura Eutin, Yanneck Sonnenberg, Nele Wachtel, Bernhard Langer

**Affiliations:** Department of Health, Nursing, Management, University of Applied Sciences Neubrandenburg, 17033 Neubrandenburg, Germany; christiankunow@googlemail.com (C.K.); gp20191@hs-nb.de (M.A.B.); gp20212@hs-nb.de (L.D.); gp20192@hs-nb.de (L.E.); gp20196@hs-nb.de (Y.S.); gp20213@hs-nb.de (N.W.)

**Keywords:** non-prescription medicines, emergency contraception, community pharmacies, information gathering, availability, pricing, mystery calls, ulipristal acetate, levonorgestrel, Germany

## Abstract

To prevent unwanted pregnancies, oral emergency contraception (EC) with the active ingredients levonorgestrel (LNG) and ulipristal acetate (UPA) is recommended by the guidelines of the German Federal Chamber of Pharmacists (BAK). In this respect, community pharmacies (CPs) in Germany have a major responsibility for information gathering, selecting the appropriate medicine, availability and pricing, among other things. Therefore, it would be appropriate to conduct a study with the aim of investigating information gathering, a possible recommendation as well as availability and pricing for oral EC in German CPs. A representative nationwide observational study based on the simulated patient methodology (SPM) in the form of covert mystery calls will be conducted in a random sample of German CPs stratified according to the 16 federal states. Each selected CP will be randomly called once successfully by one of six both female and male trained mystery callers (MCs). The MCs will simulate a product-based scenario using the request for oral EC. For quality assurance of the data collection, a second observer accompanying the MC is planned. After all mystery calls have been made, each CP will receive written, pharmacy-specific performance feedback. The only national SPM study on oral EC to date has identified deficits in the provision of self-medication consultations with the help of visits in the CPs studied. International studies suggest that UPA in particular is not always available. Significant price differences could be found analogous to another German study for a different indication.

## 1. Introduction

To prevent unwanted pregnancies, the World Health Organization (WHO) recommends the use of emergency contraception (EC). The WHO distinguishes between the copper intrauterine device (Cu IUD) for insertion into the cavum uteri, the oral EC with the active ingredients levonorgestrel (LNG) and ulipristal acetate (UPA) and the combined oral contraceptives (COCs, Yuzpe method) [1], which are not recommended in Germany, as fewer adverse effects and higher clinical efficacy were associated with LNG and UPA [2,3,4]. In Germany, the Cu IUD is a prescription-only medicine (POM), whereas the oral EC—analogous to many other countries worldwide [5]—has been available since March 2015 as an over-the-counter (OTC) medicine without prescription [6,7]. In contrast, however, there are still a number of countries, such as South Korea, where oral EC is only available with prescription [5]. In this context, Poland plays a special role, as UPA was also available as an OTC medicine as of 2015, analogous to Germany, but was made subject to prescription again in 2017 despite controversial discussions and protests [8,9]. Oral EC may only be dispensed by community pharmacies (CPs) in Germany [10]. German CPs, therefore, have a great responsibility with regard to availability and pricing as important criteria for unhindered access [11,12]. With regard to the availability, this is particularly important in view of the fact that the effectiveness of oral EC is higher the faster it is taken [1]. With regard to the pricing, this is due to the fact that the German CPs are free to set the price of oral EC as an OTC medicine since the abolition of price maintenance in 2004 [13]. The actual prices for oral EC of the individual CPs are not available online, raising the question of what prices are actually charged by individual CPs. This lack of price transparency is one of the main reasons for price differences [14], especially as the prices are also usually only disclosed on-site at the CP at the end of the dispensing process [15].

German CPs dispensed 877,000 packs of oral EC in 2019, an increase of about 32% compared to the year of the OTC switch in 2015 (662,000 packs). Oral EC packs without prescription accounted for a share of about 71% of all oral EC packs in 2015 and even about 94% in 2019 [16]. Since more and more patients want to receive oral EC without a prior visit to their doctor, German CPs are also playing an increasingly important role in the provision of self-medication consultations. According to the guidelines of the German Federal Chamber of Pharmacists (BAK) on self-medication [17], the provision of self-medication consultations represents a multi-stage process from information gathering, selecting the appropriate medicine to giving advice in the context of dispensing. In principle, CPs in Germany have to ensure “adequate” provision of self-medication consultations. The provision of self-medication consultations must be carried out by a pharmacist, but can also be carried out by non-pharmacists (e.g., pharmacy technicians and pharmaceutical technical assistants) if the pharmacy manager has previously determined this in writing [18]. The BAK has issued corresponding guidelines for the provision of self-medication consultations for oral EC—first in 2015 [19] and last updated in 2020 [20]. In addition to giving advice, which should be applicable by the pharmacy staff in the respective conversation with the persons concerned, these guidelines also contain a checklist. This checklist should be available in CPs as a physical printed or digital version to ensure that the pharmacy staff asks the people concerned questions that are relevant for a possible recommendation of oral EC. In addition to the knowledge of the pharmacy staff, which is needed anyway, surveys have found knowledge deficits and incorrect knowledge about oral EC in adolescents [21,22,23], adults [24,25,26,27] and across populations [28,29] in Germany, especially with regard to the mechanism and period of time of action. This further underlines the importance of information gathering by the pharmacy staff including a possible recommendation of oral EC.

Unlike other countries such as the USA [11,12,30,31,32,33,34,35,36,37,38,39,40,41,42,43,44,45,46,47], the study situation for Germany for the provision of self-medication consultations, availability and pricing is rather poor so far. In nationwide interviews of 25 CPs conducted at the end of 2015, i.e., after the OTC switch in March 2015, 96% of the respondents reported using a checklist for the provision of self-medication consultations for oral EC. Of these, 52% said they worked with the checklist of the BAK. In addition, 96% of all interviewees stated that they had both LNG and UPA available [48]. In contrast to the dispensing recommendation of the BAK guidelines [20], a non-representative survey of 143 CPs in Hesse showed that oral EC is not always dispensed for women with the experience of sexual violence. Analogous results were found for women with poor German language skills [49], although this group is not explicitly mentioned in the BAK guidelines [20]. In a nationwide interview study, 12 female EC users interviewed wished, among other things, for more discretion and more patient-oriented information gathering [50]. In contrast, a non-representative online survey of 555 CPs concluded, among other things, that pharmacy staff refer women to gynaecologists in the case of safety concerns [51]. In contrast, however, no studies on pricing from German CPs to oral EC are known.

With regard to the methodology to be applied, however, the disadvantage of self-reported surveys and interviews—such as the previously presented studies on the practices of German CPs on oral EC—is that the validity of the study results could be limited due to social desirability bias, as the interviewed or surveyed pharmacy staff in particular tend to present their provision of self-medication consultations better than they actually provided them [52,53]. In the case of non-participant observations, the disadvantage is that pharmacy staff usually adjust their behaviour when they realise that they are being observed (“Hawthorne effect” [54]). To avoid the problems described above, the simulated patient methodology (SPM) is recommended [55,56] in the international literature as the “gold standard” [57]—also taking into account the relatively high administrative and financial effort as well as comparatively small sample sizes [57] and any intra- and inter-observer variabilities [58]—with which a lifelike conversational situation can be depicted [59]. However, only one SPM study on oral EC is known for Germany [60], which investigated the provision of self-medication consultations for oral EC, but neither availability nor pricing, and is also a representative analysis for only one federal state. Therefore, it would be appropriate to conduct a representative nationwide study with the following objectives:

- Primary objective: to investigate information gathering based on the BAK checklist, a possible recommendation as well as availability and pricing for oral EC.
- Secondary objective: to determine to what extent the study results differ with regard to possible influencing factors.

This study is planned on the basis of the present protocol. There is already a protocol on oral EC from Australian CPs, but it is based on interviews [61].

## 2. Materials and Methods

### 2.1. Study Design

The planned study is to be based on a cross-sectional design, conducted with the help of the SPM in the form of covert mystery calls and reported in accordance with the ‘STROBE Statement—Checklist of items that should be included in reports of cross-sectional studies’ [62]. Against the background of a nationwide study, calls are to be preferred to visits—which have already been used in German CPs [15,60,63,64], but only in relation to one city or one federal state—as the implementation of calls without the requirement of a physical presence in the CP is less costly and thus more feasible. Following the international literature [59,65,66,67], the SPM in the form of covert mystery calls is a covert participant observation

- by a person (**mystery caller** (MC)),
- who contacts a **CP**,
- with the help of a **call**,
- to simulate a lifelike conversation situation based on a predefined **scenario**.

This is followed by

- the **data collection** according to predefined criteria using an **assessment form** and
- the **data management and analysis**.

In addition,

- the CP contacted is given **performance feedback**, if applicable.

### 2.2. Mystery Caller

To conduct such a study, at least 1 person is needed as MC. In order to achieve generalisable and standardisable study results, more than 2 both female and male persons [65] should be recruited. However, since Watson et al. do not specify a precise upper limit [65], the use of 13 persons could be determined on average on the basis of a current SPM systematic review [66]. Although the guidelines of the BAK only refer to the provision of self-medication consultations for women [20]—in contrast to the Australian guidelines, for example [68]—the behaviour of the pharmacy staff should also be examined with men and thus with the help of a supposedly atypical selection for such a conversation situation. However, it is quite realistic to assess when a man calls for his wife or girlfriend and wants to take the call for her because she feels uncomfortable, ashamed or might even already be psychologically burdened [69,70,71]. In a German study, more than 82% of the CPs interviewed also considered it a problem that oral EC was not requested by the woman concerned, but by the respective man or a third person [51].

Since a former student (CK) had agreed to be a male MC before the research project was announced, female MCs in particular had to be acquired. In principle, this acquisition was promising, since previous research projects of the project leader (BL) usually involved more female students due to the health and care-related Master degree programmes. Finally, 1 male and 4 females could be acquired as student MCs, who with an age between 20 and 30 years are in the age range of average users of oral EC in Germany [25,72,73]. Thus, the now total of 6 acquired MCs—including the male non-student MC (age 38)—can contribute to the simulation of a lifelike conversation situation. The MCs participate in the project free of charge.

### 2.3. Setting and Participation

The planned study is to be carried out within the framework of a 3-semester research project of various Master degree programmes in the Faculty of Health, Nursing, Management of the University of Applied Sciences Neubrandenburg from the beginning of October 2020 until the end of February 2022. In order to be able to plan a representative nationwide study, a list of all CPs registered in Germany is first necessary to determine the basic population. The free pharmacy finder of the “Apotheken Umschau” [74] was used to create a list. Thus, information from the pharmacy finder with regard to name, postcode and location of all CPs in Germany has been extracted into an MS Excel file in December 2020. To check the accuracy, the total number of 18,777 CPs thus identified was compared with the latest available total number of 19,075 CPs provided annually by the Federal Union of German Associations of Pharmacists (ABDA) and most recently for the reference date 31 December 2019 [16]. Due to the slightly decreasing number of CPs in recent years [16], the population size determined should correspond to a fairly current status.

In Germany, there are no studies on availability and pricing of oral EC. Therefore, the degree of variability is unknown. The minimum necessary sample size (*n*) was determined for the corresponding population size (*N*) and an error margin (*e*) of 0.05 using the following formula based on a degree of variability of *p* = 0.5 and a 95% confidence interval [75]:

$$n=\frac{N}{1+N{\left(e\right)}^{2}}=\frac{18,777}{1+18,777{\left(0.05\right)}^{2}}=\frac{18,777}{47.9425}=391.66$$

The assumed degree of variability of *p* = 0.5 maximises the required sample size. The 18,777 CPs were stratified by location as an indicator for the respective German federal state and assigned a random number using the MS Excel random number generator. A simple random sample was then drawn in each stratum to the extent of that stratum’s share of all CPs to select the required 392 CPs. To validate the selected CPs, a Google search was conducted and, if not already available, the telephone number was located. If, contrary to expectations, CPs are closed or cannot be found, it is planned to replace them by drawing more CPs in the corresponding stratum.

The distribution of the selected CPs to the MCs is to be done by means of the random principle, so that 65 to 66 CPs (392 CPs/6 MCs = 65.3 CPs) are assigned to each of the 6 MCs. Each of the selected CPs is to be called once successfully, so that in total there are also 392 calls (6 MCs × 65.3 CPs = 392 calls). The calls are to be made on different days of the week and at different times of the day. No costs are calculated for the execution of the calls, since all MCs have a telephone flat rate and the corresponding monthly basic fees for the MCs are incurred anyway.

In addition, 3 months before the start of the main study, 5 test calls (30 calls in total) will be made by each of the 6 MCs to CPs outside the random sample as part of a pilot study. This is to test the functionality of the SPM planned here in order to identify possible weaknesses. The MCs will conduct the test calls from home, but the project leader will be available at any time via a video conferencing system and the MCs are also required to discuss any problems that arise immediately together with the project leader in order to adjust the study protocol accordingly. These test calls also serve as practical training for the MCs, who have already familiarised themselves with the theoretical basics of the SPM and conducted a role play. After the role plays and the test calls, there will be a workshop to exchange experiences and inform each other about the special features of the scenario and the assessment form. If necessary, the scenario and the assessment form will be adapted accordingly.

### 2.4. Scenario

**Figure 1:**
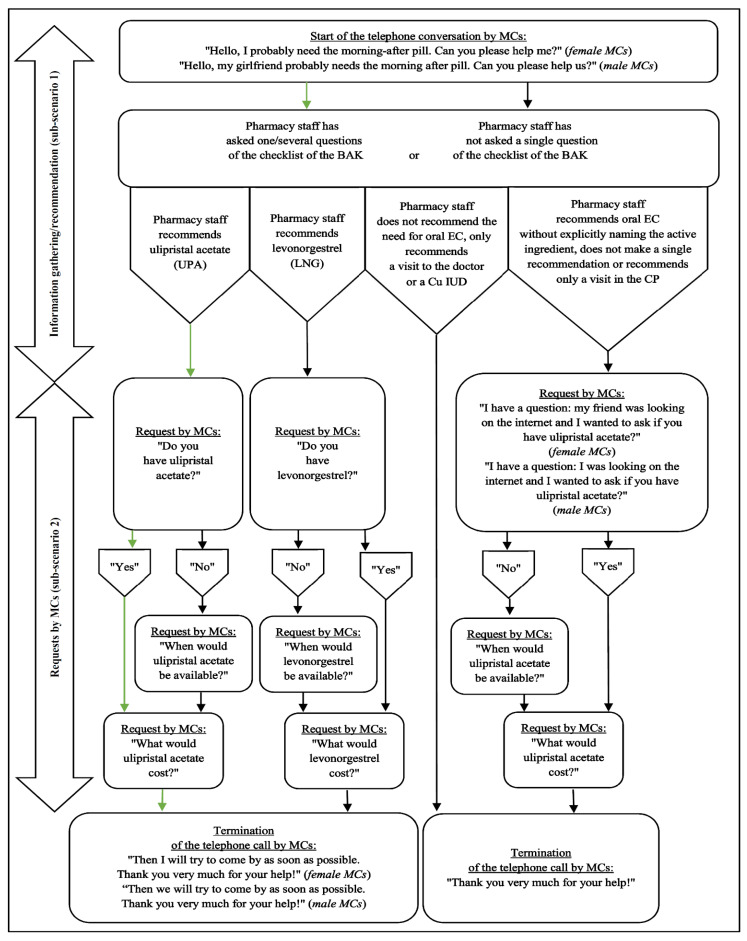
Scenario for female and male MCs using a flow chart. Note: The green arrows indicate the most optimal course of conversation.

The conversation situation to be simulated, which should be as true to life as possible, should be based on a product-based scenario (Figure 1). The reason for planning a product-based rather than a symptom-based scenario is that the availability, pricing and provision of self-medication consultations for oral EC should be examined and the pharmacy staff should be specifically directed towards this. Basically, the scenario should only differ in the use of a female or male MC, i.e., in gender-specific answers or questions from the MCs.

The MCs should start the telephone conversation by saying that they probably need oral EC and then ask if the pharmacy staff can help. Thus, the person concerned should be disclosed at the beginning of the conversation. The disclosure of this rather little information at the beginning of the conversation can contribute to more comprehensive information gathering, since the pharmacy staff has to gather more information, e.g., about the reason for the call, by asking. In addition, the aim is to signal to the pharmacy staff with the word “probably” uncertainty about the need for oral EC and with the word pair “please help” a request for help for the immediate initiation of information gathering on the phone. From this point on, the pharmacy staff could want to end the conversation at any time and refer the supposedly affected person to a visit in the CP or a doctor’s visit, for example. It is not necessary for MCs to ask for a pharmacist at the beginning of the conversation, as in Germany the provision of self-medication consultations is also possible through non-pharmacists.

If the pharmacy staff were to start information gathering, they could ask one or more questions from the BAK checklist [20]. The questions listed in Table 1 correspond completely to the questions of the BAK checklist relevant for everyday information gathering in Germany. Since one of our study objectives is to investigate the extent to which CPs ask questions from the BAK checklist, only these questions should be considered in the scenario. Therefore, further questions resulting from international guidelines (e.g., on weight) [76] should not be included in the scenario.

In connection with information gathering based on the checklist of the BAK [20], there should be corresponding answer guidelines for these questions for the MCs (Table 1), including that the girlfriend is 24 years old. Since it does not seem so realistic that the girlfriend has talked to the boyfriend about the strength, length and unusualness of the menstrual period (5th to 8th question) in the run-up to the call, the male MCs should answer “I don’t know” if the pharmacy staff should ask about this. In principle, the answers of the MCs should be given in such a way that the conversation is quite easy for the MCs to simulate and the information gathering should be quite uncomplicated for the pharmacy staff. However, the pharmacy staff could also recommend only UPA, only LNG, UPA or LNG alternatively, the Cu IUD, oral EC without explicitly naming the active ingredient, or the need for no oral EC, regardless of whether information gathering is carried out appropriately. Otherwise, or in addition, the pharmacy staff could recommend a doctor’s visit or a visit to the CP (sub-scenario 1).

After the possible information gathering activities and the recommendation of UPA or LNG, the MCs should ask follow-up questions: about availability and the costs of the recommended oral EC. If the pharmacy staff recommends the need for no oral EC, only a visit to the doctor or a Cu IUD, the MCs should not doubt the respective recommendation and end the conversation. If the pharmacy staff recommends oral EC without explicitly naming the active ingredient, does not make a single recommendation or only recommends a visit to the CP, the MCs should ask about the availability and the costs of UPA specifically, stating that their friend or they have informed themselves on the internet (sub-scenario 2).

Afterwards, the MCs are supposed to end the conversation—also according to the respective course—by thanking the pharmacy staff for the help and saying that they will try to come by as soon as possible. That the MCs should be given this statement is based on the fact that the use of the previously recommended oral EC is recommended as soon as possible after the unprotected sexual intercourse (UPSI) due to the greater effectiveness [20] and that the pharmacy staff might have given such a hint beforehand. If they have recommended the need for no oral EC, only a doctor’s visit, a Cu IUD, oral EC without explicit mention of the active ingredient, only a visit in the CP or have not made a single recommendation, the MCs should only thank them for their help at the end.

### 2.5. Assessment

Analogous to the planned scenario, the items for the assessment should be based on the questions of the BAK checklist [20] and the possible recommendation (sub-scenario 1) as well as on the answers of the pharmacy staff on availability and pricing (sub-scenario 2). Since oral EC cannot be dispensed over the phone and the giving advice associated with dispensing usually takes place on-site at the CP, no items were collected for giving advice. Influencing factors obtained from the literature shall complete the assessment (Table 2). The planned items shall only be objective and mostly use dichotomous scales (closed yes/no questions).

In connection with the checklist and the guidelines of the BAK [20], the question of the age of the woman concerned (item 1) therefore plays a role, as the pharmacy staff should question the necessity of oral EC if the age is outside the childbearing age. In addition, dispensing oral EC to girls under 14 years of age without the consent of a legal guardian is not recommended [20]. The question about the reason for oral EC (item 2), on the other hand, is of central importance, since the pharmacy staff should only recommend oral EC if a UPSI has taken place [20]. The question of the timing of the UPSI (item 3) plays an even greater role here, since UPA and LNG are only effective in a certain period after UPSI [20]. In principle, UPA has the larger temporal window of effect, whereby UPA has also been shown to be more effective than LNG in terms of pregnancy rates in the first 24 or 72 h after the UPSI [77,78,79]. The guidelines of the BAK [20] advise the pharmacy staff, if the UPSI is no more than 72 h (3 days) ago, to recommend LNG or UPA to the person, if more than 72 h but no more than 120 h (5 days) ago, to recommend UPA exclusively. If, on the other hand, the UPSI occurred more than 120 h ago, a visit to a gynaecologist should be recommended instead of oral EC [20].

The questions about the last menstrual period (item 4–8) could give the pharmacy staff clues about an existing pregnancy, after which they should not recommend oral EC according to national guidelines [20], although it does not harm an existing pregnancy according to international guidelines [76]. The question about acute health problems or chronic diseases (item 9) is relevant because the pharmacy staff should recommend UPA in the case of an increased risk of thrombosis and the case of severe liver dysfunction a further visit to the doctor in addition to dispensing oral EC [20]. The question as to whether breastfeeding is taking place (item 10) is primarily aimed at information to be given by the pharmacy staff regarding a break from breastfeeding. For UPA, both national [20] and international guidelines [76] recommend a breastfeeding break of 1 week. In contrast, international guidelines [76] do not impose any restrictions for LNG, whereas national guidelines [20] recommend breastfeeding immediately after ingestion and a subsequent breastfeeding break of 8 h, as LNG passes into breast milk. The question about the use of medicines (item 11) is relevant because the pharmacy staff should point out that the effectiveness of UPA and LNG may be reduced if certain medicines are taken at the same time [20]. In addition, the question about the use of oral EC in the past (item 12) is relevant because, according to the national guidelines [20], repeated use of LNG within the same menstrual cycle should not be recommended by the pharmacy staff, whereas the international guidelines [76] do not make this restriction.

Based on the answers of the respective MC to the questions asked (item 1–12), the pharmacy staff should make a recommendation regarding EC (item 13–17) and recommend UPA (“appropriate outcome”). Since the MCs should not give any information in their answers about, for example, existing interactions or contraindications, the pharmacy staff could make this recommendation solely on the basis of the answer (“4 days ago.”) to the question about the time of the UPSI (item 3). A recommendation for a visit to the doctor (item 18) would only make sense if a Cu IUD was recommended at the same time, which would not be effective in the planned scenario and would probably cost valuable time to prevent an unwanted pregnancy. A recommendation of a visit to the CP (item 18) would only make sense if UPA was recommended at the same time, since the person concerned would have to come to the CP to buy it anyway. In principle, the recommendation of a visit to the CP would be welcome, but only if it is clarified in the telephone consultation whether oral EC is necessary at all. Otherwise, an unnecessary journey would be imposed on the person concerned.

In addition, the (recommended) oral EC (item 19) should ideally be available immediately when asked about availability. If the oral EC is not available, it would be welcome from a service point of view if the pharmacy staff informs which CP could have it available (item 19a). In addition, if the time of availability is requested (item 20a), it should be available on the same day, since with a (stated) UPSI before 4 days, those affected would only have a few hours to receive the oral EC and then take it in order to be able to prevent an unwanted pregnancy. If LNG is recommended instead, its immediate availability (item 20b) would be welcomed in principle, but would not be helpful in this planned scenario, since according to the guidelines of the BAK [20], LNG should only be given up to 72 h (3 days) after UPSI. In connection with the assessment of the (lowest) indicated price of the (recommended) oral EC (item 21), it should be noted that in Germany—in contrast to LNG—there are currently significantly fewer preparations available on the market [80], so that there is less choice.

Influencing factors to be investigated include:

- the MC number (item 22) [81],
- the gender of the MC (item 23) [82],
- the gender of the pharmacy staff (item 24) [83], which is usually identifiable by the voice during the call,
- a possible quality certificate of the CP (item 25) [84], which should be determined by the MC on the same day of the call on the internet—if documented there—and which—if not yet determined—should be asked for on the basis of a further call after all calls have been completed
- as well as the length of the telephone call (item 26) [85] by using a clock accurate to the second.

In addition, questions or statements made by the pharmacy staff outside the planned scenario will be identified (item 27).

### 2.6. Data Collection

The assessment items will be transferred to an assessment form (Table 2) for data collection. In addition to the items, the call attempts are to be collected. In this regard, the MCs should try to reach the CP via their private mobile phone with a suppressed number a maximum of 3 times spread over a single possible day. If a CP could not be reached, the MCs shall mark the CP as unavailable, thus replacing it by drawing another CP in the corresponding stratum. If the call is held in a queue for at least 15 min, the MCs shall hang up and call again a little later on the same day. If an answering machine picks up the call, the MCs should hang up immediately and try calling again a little later on the same day. If the pharmacy staff inadvertently hangs up before the call is completed, the MCs should call back immediately. If the call is interrupted due to reception problems or if the MCs forget to ask questions before finishing the call, the MCs should also call back immediately. If the MCs suspect or are certain that their call has been discovered, the respective CP shall be replaced by drawing another CP in the corresponding stratum.

No (covert) audio recordings will be made during the calls for quality assurance purposes, as otherwise the corresponding consent of the CPs would have to be obtained in advance [86], which would, however, give the possibility of not participating in the study (opt-out), which in turn could lead to a selection bias and thus to a biased assessment [87,88]. Due to the detailed scenario, however, the use of a second observer is planned for each call, who should listen in on the MC’s call via the loudspeaker or telephone conference function of the MC’s mobile phone. One second observer will come from the family environment of one of the MCs and will be trained in the same way as the MCs. The other second observers will come from the group of the other MCs and will therefore already be trained. The MC should then complete the assessment form in writing immediately after the call with the help of the second observer, who should take notes during the call. Possible disagreements should be clarified by a discussion between the MC and the second observer. In case of unsuccessful clarification, the assessment of the second observer is decisive.

As CPs are classified as systemically relevant in Germany and therefore remain open [89], the COVID-19 pandemic should not have any impact on the implementation of the planned data collection.

### 2.7. Data Management and Analysis

The data are to be entered using the “four-eyes principle” (one MC enters the data, while the second observer checks the quality of the data entry) and analysed with SPSS version 26 for Windows (IBM, Armonk, NY, USA). Within the framework of descriptive statistics, frequencies and percentages are to be determined for categorical data. In addition, 95% confidence intervals will be reported for categorical data using bootstrapping. With the help of the Shapiro–Wilk test as well as the Kolmogorov–Smirnov test, it is to be tested for continuous data whether they are normally distributed. In the case of a normal distribution, the mean, standard deviation, minimum and maximum as well as the range should be reported, whereas, in the case of non-normally distributed data, the median, interquartile range, minimum and maximum as well as the range should be presented [90]. Since unconnected samples are involved, a chi-square test (or alternatively an exact test according to Fisher for expected cell frequencies below five) should be applied for categorical variables to determine correlations. Cramer’s V should be reported as an effect size measure, whereby, according to Cohen, a small effect exists from 0.10, a medium effect from 0.30 and a large effect from 0.50 [91]. For continuous data, the *t*-test for unconnected samples should be used in the case of a normal distribution, and the Mann–Whitney U-test should be used in the case of non-normally distributed data to determine differences between the groups. If the *t*-test is used for independent samples, Cohen’s d (from 0.20 a small effect, from 0.50 a medium effect and from 0.80 a large effect) should be used as an effect size measure [91]. If the Mann–Whitney U-test is used, the effect size should be measured with the help of the Pearson correlation coefficient r, whereby, according to Cohen, there is a small effect from 0.10, a medium effect from 0.30 and a large effect from 0.50 [91]. In all statistical analyses, a *p*-value less than 0.05 should be considered significant.

### 2.8. Performance Feedback

After evaluation of the data—as recommended internationally [59,66]—each CP should receive written, pharmacy-specific performance feedback including graphically prepared benchmarking by e-mail or letter post, whereby the improvement or deterioration with regard to the individual items is shown for each CP in comparison to the remaining, anonymously presented CPs. This provides the CPs with information about their competitive position, so that ideally—if necessary—corresponding optimisation processes can be initiated on the part of the examined CPs with the aim of sustainably improving information gathering and selecting the appropriate medicine. It would be ideal if these optimisation processes would be initiated and accompanied by the research team, but this is not possible due to time and financial restrictions. In addition, it is planned to provide the CPs with general performance feedback on the basis of the planned publication of the study results. In a German SPM study, it was reported that feedback was well accepted by the pharmacy staff [92].

### 2.9. Ethics and Dissemination

The study planned here has been applied for and approved by the Ethics Committee of the University of Applied Sciences Neubrandenburg, Germany (protocol code HSNB/171/21). According to the “Guideline for the Use of Mystery Research in Market and Social Research” [93], the data are processed in such a way that neither the CPs involved in the study nor their personnel can be identified. This applies to both the data collection and storage as well as the publication of the research results in a peer-reviewed journal. There is also no picture or sound recording of the calls. This ensures that the pharmacy owner or its staff are not exposed to any criminal or civil liability or that their reputation is damaged by the investigation. Nevertheless, in order to meet the CPs’ need for information, a letter was sent to all participating CPs in March 2021—analogous to recommendations in the international literature [87,88] and to the implementation in numerous studies [94,95,96]—informing them about the background and the conduct of the study. However, in order not to jeopardise the covert study design, a correspondingly long time period (calls are planned from August to October 2021) was specified in this letter instead of a specific date, in that calls will be conducted at a time unknown to the CPs. In addition, it is planned that the persons recruited as MCs and second observers will sign a declaration stating that they agree to act as MCs and second observers, respectively.

## 3. Discussion

The study planned here is of enormous importance to answer the questions whether the German CPs offer information gathering including a possible recommendation of oral EC on the phone, and whether they live up to their great responsibility for continuous availability and relatively accessible prices without too great price differences. Ideally, these framework conditions should be in place so that, in such an emergency, those women are given the opportunity to prevent an unwanted pregnancy. In addition, representative nationwide results would considerably improve the rather poor study situation in Germany.

It will be interesting to see to what extent information gathering or a possible recommendation of oral EC takes place on the phone or is referred to a visit to the CP. In any case, the only national SPM study on oral EC to date has identified deficits in the provision of self-medication consultations with the help of visits in the CPs studied [60]. Regarding the availability of oral EC, international SPM studies [37,38,39,41,42,45] suggest that there are problems—especially with UPA. Some price differences—even between identical preparations—could also be identified, as in addition to international SPM studies on oral EC [11,12,41,42,43,44,45,46,47,97,98], a national SPM study on a different indication has already identified significant price differences between CPs that are even located in the same city and in some cases only a few hundred metres apart [15].

### Strengths and Limitations

As far as the authors are aware, this will be the first representative nationwide study in Germany to investigate oral EC practices including information gathering, a possible recommendation, availability and pricing of CPs with the help of the internationally already frequently used [59,65,66] SPM in the form of covert mystery calls. However, as the study is planned with a cross-sectional design, interpretations of the results will be limited, i.e., no causal relation between studied variables can be established. In order to be able to determine how results change over time, it would make more sense to plan a longitudinal study, but, due to the high financial and time costs involved, we refrain from doing so.

The information provided by the pharmacy staff may also differ in calls from face-to-face situations. For example, although the actual prices are to be determined, different prices may be quoted on the phone than on the spot. Since certain MCs were recruited to carry out the planned study and are in a certain age range, it cannot be ruled out that information gathering including a possible recommendation could turn out differently for those affected from other educational strata or age groups [98]. Furthermore, other scenarios could lead to different results [99,100,101]. However, the scenario planned here is very comprehensive analogous to other international EC studies using a MC approach [33,34,100]. Finally, the validation based on the pilot study could have been additionally planned with other persons than the acquired MCs, which could probably improve the approach [102].

With regard to the assessment, no items are planned that could allow a subjective assessment and thus a margin of discretion in the assessment (e.g., the friendliness of the pharmacy staff). Since a second observer is planned for the quality assurance of the data collection, distortions in the study results due to possible lacking or faulty memories (recall bias) of the MCs should be minimised. Finally, pharmacy-specific performance feedback would be desirable directly after the respective call, since the pharmacy staff’s memory of the specific conversation situation should be most present at that time [59]. However, there is a risk that the pharmacy staff will inform other CPs in the vicinity about the call and then the subsequent calls can only be evaluated in a distorted way.

## Figures and Tables

**Table 1:** Sub-scenario 1 for female and male MCs (without the possible recommendation).

Possible Questions by the Pharmacy Staff Based on the Questions of the BAK Checklist [20]	Response Specifications for MCs
**1.** “How old are you?” “How old is your girlfriend?”	“I am 24.” (female MCs) “She’s 24.” (male MCs)
**2.** “Why do you need oral EC?”	“We had a condom failure.”
**3.** “When was the unprotected sexual intercourse?”	“4 days ago.”
**4.** “When was your last menstrual period?”	“11 days ago.”
**5.** “Is the date of the first day of the last menstrual period more than 28 days ago?” **6.** “Was your last menstrual period weaker than usual?” **7.** “Was the last menstrual period shorter than usual?” **8.** “Was the last menstrual period unusual in any other way?”	5th to 8th: “No.” (female MCs) 5th to 8th: “I don’t know.” (male MCs)
**9.** “Are you aware of any acute health problems or chronic illnesses?”	“No.” (female MCs)
“Is your friend aware of any acute health problems or chronic illnesses?”	“No.” (male MCs)
**10.** “Are you currently breastfeeding?” “Is your girlfriend currently breastfeeding?”	“No.” (female MCs) “No.” (male MCs)
**11.** “Are you currently taking any medication?” “Is your friend currently taking any medication?”	“No.” (female MCs) “No.” (male MCs)
**12.** “Have you ever used oral EC before?” “Has your friend ever used oral EC?”	“No.” (female MCs) “No.” (male MCs)

**Table 2:** Assessment items for female and male MCs.

Information Gathering Including a Possible Recommendation of Oral EC by Pharmacy Staff (Based on Sub-Scenario 1)		
**1.** Did the pharmacy staff ask for the age?	Yes ☐	No ☐
**2.** Did the pharmacy staff ask the reason for the request for oral EC?	Yes ☐	No ☐
**3.** Did the pharmacy staff ask for the time of the UPSI?	Yes ☐	No ☐
**4.** Did the pharmacy staff ask for the time of the last menstrual period?	Yes ☐	No ☐
Did the pharmacy staff enquire whether… **5.** … the date of the first day of your last menstrual period was more than 28 days ago? **6.** … the last menstrual period was weaker than usual? **7.** … the last menstrual period was shorter than usual? **8.** … the last menstrual period was otherwise unusual?	Yes ☐ Yes ☐ Yes ☐ Yes ☐	No ☐ No ☐ No ☐ No ☐
**9.** Did the pharmacy staff ask whether any acute health problems or chronic illnesses are known?	Yes ☐	No ☐
**10.** Did the pharmacy staff ask if you are currently breastfeeding?	Yes ☐	No ☐
**11.** Did the pharmacy staff ask whether any medicines are being taken?	Yes ☐	No ☐
**12.** Did the pharmacy staff asked whether the morning-after pill has ever been used?	Yes ☐	No ☐
**13.** Did the pharmacy staff recommend UPA? **14.** Did the pharmacy staff recommend LNG? **15.** Did the pharmacy staff recommend Cu IUD? **16.** Did the pharmacy staff recommend oral EC without naming the active substance? **17.** Did the pharmacy staff recommend not needing oral EC?	Yes ☐ Yes ☐ Yes ☐ Yes ☐ Yes ☐	No ☐ No ☐ No ☐ No ☐ No ☐
**18.** What else did the pharmacy staff recommend?	Visit to the doctor ☐ Visit in CP ☐	
**Requests by MCs (Based on Sub-Scenario 2)**		
**19.** Did the CP have oral EC available? **19.a.** Did the pharmacy staff inform what CP might have the oral EC available?	Yes ☐ (UPA) Yes ☐ (LNG) Yes ☐ (UPA) Yes ☐ (LNG)	No ☐ (UPA) No ☐ (LNG) No ☐ (UPA) No ☐ (LNG)
**20.a.** When would the CP have UPA available? **20.b.** When would the CP have LNG available?	on the same day ☐ the next day ☐ later than the next day ☐ unknown ☐ on the same day ☐ the next day ☐ later than the next day ☐ unknown ☐	
**21.** What is the (lowest) price quoted by the pharmacy staff for oral EC?	Price (UPA): … Price (LNG): …	
**Possible Influencing Factors**		
**22.** MC number?	…	
**23.** What is the gender of the MC?	female ☐	male ☐
**24.** What is the gender of the pharmacy staff?	female ☐	male ☐
**25.** Does the CP have a quality certificate?	Yes ☐ No ☐ unknown ☐	
**26.** How long did the telephone conversation last?	…, … min.	
**27.** Did the pharmacy staff ask questions or make statements that are not planned in the scenario? If “Yes”, which ones?	Yes ☐	No ☐
	……………………………………	

Note: The possible influencing factors were taken from the specific literature sources cited in the manuscript.

## Data Availability

All of the study data will be available to interested researchers upon request to Bernhard Langer, who is responsible for the project. Requests will be reviewed by the research team and will require a data transfer agreement.
